# Association of metabolic dysfunction-associated fatty liver disease with systemic atherosclerosis: a community-based cross-sectional study

**DOI:** 10.1186/s12933-023-02083-0

**Published:** 2023-12-13

**Authors:** Yanli Zhang, Zhang Xia, Xueli Cai, Xin Su, Aoming Jin, Lerong Mei, Jing Jing, Suying Wang, Xia Meng, Shan Li, Mengxing Wang, Tiemin Wei, Yongjun Wang, Yan He, Yuesong Pan

**Affiliations:** 1 Department of Neurology, Beijing Tiantan Hospital, Capital Medical University, No.119, South 4th Ring West Road, Fengtai District, Beijing, 100070 China; 2 China National Clinical Research Center for Neurological Diseases, Beijing, China; 3 Department of Epidemiology and Health Statistics, School of Public Health, Capital Medical University, Beijing, China; 4 Beijing Municipal Key Laboratory of Clinical Epidemiology, Beijing, China; 5 Department of Neurology, The Fifth Affiliated Hospital of Wenzhou Medical University, Lishui, China; 6 Cerebrovascular Research Lab, The Fifth Affiliated Hospital of Wenzhou Medical University, Lishui, China; 7 Department of Cardiology, The Fifth Affiliated Hospital of Wenzhou Medical University, Lishui, China; 8 Advanced Innovation Center for Human Brain Protection, Capital Medical University, Beijing, China; 9 Research Unit of Artificial Intelligence in Cerebrovascular Disease, Chinese Academy of Medical Sciences, Beijing, China; 10 Center for Excellence in Brain Science and Intelligence Technology, Chinese Academy of Sciences, Shanghai, China

**Keywords:** Fatty liver disease, Metabolic dysfunction, Atherosclerosis, Polyvascular disease, Diabetes mellitus

## Abstract

**Background:**

Data are limited on the association of metabolic dysfunction-associated fatty liver disease (MAFLD) with systemic atherosclerosis. This study aimed to examine the relationship between MAFLD and the extent of atherosclerotic plaques and stenosis, and presence of polyvascular disease (PolyVD).

**Methods:**

In this cross-sectional study, MAFLD was diagnosed based on the presence of metabolic dysfunction (MD) and fatty liver disease (FLD). MAFLD was divided into three subtypes: MAFLD with diabetes mellitus (DM), MAFLD with overweight or obesity (OW), as well as MAFLD with lean/normal weight and at least two metabolic abnormalities. Atherosclerosis was evaluated, with vascular magnetic resonance imaging for intracranial and extracranial arteries, thoracoabdominal computed tomography angiography for coronary, subclavian, aorta, renal, iliofemoral arteries, and ankle-brachial index for peripheral arteries. The extent of plaques and stenosis was defined according to the number of these eight vascular sites affected. PolyVD was defined as the presence of stenosis in at least two vascular sites.

**Results:**

This study included 3047 participants, with the mean age of 61.2 ± 6.7 years and 46.6% of male (n = 1420). After adjusting for potential confounders, MAFLD was associated with higher extent of plaques (cOR, 2.14, 95% CI 1.85–2.48) and stenosis (cOR, 1.47, 95% CI 1.26–1.71), and higher odds of presence of PolyVD (OR, 1.55, 95% CI 1.24–1.94) as compared with Non-MAFLD. In addition, DM-MAFLD and OW-MAFLD were associated with the extent of atherosclerotic plaques and stenosis, and presence of PolyVD (All *P* < 0.05). However, lean-MAFLD was only associated with the extent of atherosclerotic plaques (cOR, 1.63, 95% CI 1.14–2.34). As one component of MAFLD, FLD per se was associated with the extent of plaques and stenosis in participants with MAFLD. Furthermore, FLD interacted with MD to increase the odds of presence of systemic atherosclerosis (*P* for interaction ≤ 0.055).

**Conclusions:**

MAFLD and its subtypes of DM-MAFLD and OW-MAFLD were associated with the extent of atherosclerotic plaques and stenosis, and presence of PolyVD. This study implicated that FLD might be a potential target of intervention for reducing the deleterious effects of MAFLD on systemic atherosclerosis.

**Supplementary Information:**

The online version contains supplementary material available at 10.1186/s12933-023-02083-0.

## Introduction

Atherosclerosis is a hallmark for the development of cardiovascular diseases [1]. It was reported that nearly 94% of the older community population had the presence of atherosclerosis, and more than 80% had multivessel atherosclerotic plaques [2]. In addition, we also observed that 53% had multivessel atherosclerotic lesions among participants with cardiovascular risk factors [3]. Compared with single-site atherosclerosis, multi-site atherosclerosis was associated with a higher rate of one-year cardiovascular events, increasing from 12% (single site) to 21% (two sites) and 26% (three sites) [4]. Assessment of systemic atherosclerosis may precisely evaluate atherosclerotic vascular lesions and find potential diseases that cannot be detected when we considered only one site [5]. Therefore, risk evaluation of multi-site atherosclerosis is of importance in asymptomatic community residents.

Metabolic dysfunction-associated fatty liver disease (MAFLD) was a novel terminology proposed by the international expert panel in 2019 that highlights the coexistence of fatty liver disease (FLD) and a condition of systemic metabolic dysfunction [6–8]. Subsequent study showed that MAFLD was a more accurate and practical indicator to discriminate the risk of extra-hepatic diseases than nonalcoholic fatty liver disease [9, 10]. Recent studies reported that MAFLD was associated with an increased risk of subclinical atherosclerosis in coronary, carotid and peripheral arteries [11–13]. However, most studies focused on the relationship of MAFLD with subclinical atherosclerosis in a single or few vascular beds [11, 13, 14]. There are limited data on the association of MAFLD with multivessel atherosclerotic plaques and stenosis, which may hinder the understanding of whether MAFLD could increase the risk of cardiovascular events by inducing multi-site atherosclerosis and which subtype of MAFLD is most closely associated with multi-site atherosclerosis.

In this study, we therefore assessed the association of MAFLD with the extent of atherosclerotic plaques and stenosis, and presence of polyvascular disease (PolyVD) in community-dwelling adults.

## Materials and methods

### Study design and participants

Data were derived from the baseline survey of the Polyvascular Evaluation for Cognitive Impairment and Vascular Events (PRECISE) study. The rationale and design of the PRECISE study (NCT03178448) had been described [15]. Briefly, the PRECISE study is a community-based prospective cohort study, which used cluster sampling to enroll 3067 participants aged 50 to 75 years from six villages and four communities of Lishui city in China between May 2017 and September 2019. The exclusion criteria were as follows: contraindications for computed tomography angiography (CTA) and magnetic resonance imaging (MRI), life expectancy ≤ 4 years due to advanced cancers and other diseases, and mental diseases. The study protocol was approved by the Ethics Committees of Beijing Tiantan Hospital (IRB approval number: KY2017-010-01) and Lishui Hospital (IRB approval number: 2016-42). Informed consent was obtained from all participants prior to enrollment. This study followed the Strengthening the Reporting of Observational Studies in Epidemiology (STROBE) reporting guideline and was conducted in accordance with the declaration of Helsinki.

### Clinical and laboratory measurements

The data of clinical and laboratory measurements were collected by trained research coordinators at Lishui Hospital. Baseline demographics, lifestyle, medical history, family history, and medication use were collected with a standardized questionnaire through face-to-face interview. Height, body weight, waist circumference, and blood pressure were measured through physical examination. Body mass index (BMI) was calculated as weight (kg) divided by height (m) squared. Blood pressure was measured three times in seated position after resting for 5 min using an automated sphygmomanometer (OMRON Model HEM-7071, Omron Co.), and calculated as the average of the second and third measurements. Fasting blood samples were collected to measure blood routine indices, hepatorenal function indices, lipid profile, fasting blood glucose (FBG), glycosylated hemoglobin A1c (HbA1c), and fasting insulin. All the serum specimens were performed on the ARCHITECT c16000 auto-analyzer (Abbott, Abbott Park, Illinois, USA). FBG was measured using the hexokinase/glucose6-phosphate dehydrogenase method. HbA1c was measured by high-performance liquid chromatography. Serum triglycerides (TG), total cholesterol (TC), high-density lipoprotein cholesterol (HDL–C), and low-density lipoprotein cholesterol (LDL-C) were measured by the enzymatic colorimetric method. Estimated glomerular filtration rate (eGFR) was calculated by the Chronic Kidney Disease-Epidemiology Collaboration equation with an adjusted coefficient of 1.1 for the Asian population [16]. Homeostatic model assessment-insulin resistance (HOMA-IR) was calculated as FBG (mmol/L) multiplied by fasting insulin (mU/L), then divided by 22.5 [17].

Current smoking was defined as smoking at least one cigarette per day on average during the past month. Current drinking was defined as responding “Current alcohol consumption” to the question “Are you drinking during past 12 months?”. Diabetes was defined as FBG ≥ 7.0 mmol/L, or 2-h post-load glucose ≥ 11.1 mmol/L, or HbA1c ≥ 6.5%, or current use of antidiabetic drugs, or any self-reported history of diabetes [18]. Hypertension was defined as systolic pressure ≥ 140 mmHg or diastolic pressure ≥ 90 mmHg, or any use of antihypertensive drugs, or a self-reported history of hypertension [19]. Dyslipidemia was defined as TC ≥ 240 mg/dL, or HDL-C < 40 mg/dL, or LDL-C ≥ 160 mg/dL, or use of lipid-lowering drugs, or any self-reported history of dyslipidemia [20].

### MAFLD definition

FLD was defined as fatty liver index ≥ 30, which has 71.2% of sensitivity and 71.3% of specificity for diagnosing MAFLD [21]. MAFLD was diagnosed based on the presence of FLD plus at least one of the following three conditions: type 2 diabetes mellitus (T2DM), overweight or obesity (BMI  ≥ 23 kg/m^2^ in Asians), or lean/normal weight but presence of at least two metabolic abnormalities [22]. Metabolic abnormalities include: (1) waist circumference  ≥ 90 cm in Asian men and  ≥ 80 cm in Asian women; (2) blood pressure  ≥ 130/85 mmHg or specific drug treatment; (3) TG  ≥ 150 mg/dL or specific drug treatment; (4) HDL-C < 40 mg/dL in men and < 50 mg/dL in women or specific drug treatment; (5) prediabetes; (6) HOMA-IR score  ≥ 2.5; and (7) plasma high-sensitivity C-reactive protein > 2 mg/L [22]. MAFLD was further divided into three subtypes including DM-MAFLD (presence of FLD and T2DM), OW-MAFLD (presence of FLD and overweight or obesity without T2DM), and lean-MAFLD (presence of FLD and at least two metabolic abnormalities without overweight or obesity or T2DM) [22]. No metabolic dysfunction (MD) was defined as absence of T2DM, overweight or obesity, and less than two metabolic abnormalities. Metabolic syndrome was defined according to the criteria of International Diabetes Federation [23]. Details regarding aforementioned definitions were described in Additional file 1: Table S1.

### MRI acquisition and assessment

Intracranial and extracranial arteries were evaluated by vascular MRI on a 3.0T scanner (Ingenia 3.0T, Philips, Best, The Netherlands). MRI sequences included 3-dimensional time-of-flight magnetic resonance angiography (3D TOF MRA), 3-dimensional isotropic high-resolution black-blood T1w vessel wall imaging (3D T1w VWI), and simultaneous noncontrast angiography and intraplaque hemorrhage imaging. The segments of intracranial arteries included the internal carotid artery, M1 and M2 segments of the middle cerebral artery, A1 and A2 segments of anterior cerebral artery, P1 and P2 segments of the posterior cerebral artery, V4 segments of the vertebral artery. The segments of extracranial arteries included the proximal internal carotid artery, V1, V2 and V3 segments of the vertebral artery, and common carotid artery. The presence of atherosclerotic plaques in intracranial and extracranial arteries was defined as eccentric wall thickening with or without luminal stenosis identified on the 3D TOF MRA or 3D T1w VWI images [24]. The presence of atherosclerotic stenosis in above arteries was defined as 50–99% of stenosis or occlusion according to the Warfarin-Aspirin Symptomatic Intracranial Disease Trial and the North American Symptomatic Carotid Endarterectomy Trial (NASCET) [25, 26].

MRI data were collected and stored in the format of digital imaging and communications in medicine (DICOM) on discs and were analyzed by two raters (D.Y. and H.L.), who were blinded to participant’ information, at the Imaging Research Center of Tiantan Hospital. Final determination was further made by the third senior neurologist (J.J.) if there was any discrepancy. Cohens κ was calculated for interobserver agreement on the classification for presence of plaques and artery stenosis ≥ 50% (Cohen κ = 0.97 and 0.79 for intracranial artery and Cohen κ = 0.94 and 0.86 for extracranial artery).

### CTA acquisition and assessment

Coronary, subclavian, aorta, renal, and iliofemoral arteries were evaluated by thoracoabdominal CTA using one dual-source CT scanner (SOMATOM Force, Siemens Healthineers, Forchheim, Germany). Contrast agents iodophor (320 mg I/mL; Visipaque, GE Healthcare) was administered prior to CTA examination. CTA data were collected and stored in DICOM format on discs and were analyzed by two raters (Z.Z.Q. and Z.Z.X.), who were blinded to participant’ information, in the Core Imaging Laboratory of Keya Medical Technology (Shenzhen, China). The 3D anatomical geometry of the input CTA images was automatically reconstructed using a multitask deep learning network, and quantitative results for plaques and stenosis were calculated and characterized. Subsequently, an experienced imaging analyst identified the presence of atherosclerosis according to the 3D geometry, quantitative results, and CTA images.

The segments of coronary arteries included the left main, left descending, left circumflex, obtuse margin, diagonal, septal branch, right coronary, posterior branches, and right posterior descending segments; subclavian arteries included left and right segments; aorta arteries included arcus aortae and abdominal aorta segments; renal arteries included left and right segments; and iliofemoral arteries included common iliac, internal iliac and external iliofemoral segments. The presence of atherosclerotic plaques in coronary, subclavian, aorta, renal, and iliofemoral arteries was defined as tissue structures of at least one square millimeter area within or adjacent to the artery lumen and discernable from the vessel lumen [27]. The presence of atherosclerotic stenosis in above arteries was defined as 50–99% of stenosis or occlusion according to the Society of Cardiovascular Computed Tomography criteria [28]. Cohens κ was calculated for interobserver agreement on the classification for presence of plaques and artery stenosis ≥ 50% (Cohen κ = 0.96 and 0.92).

### ABI measurement

Peripheral arteries were evaluated by ankle-brachial index (ABI) using Doppler ultrasound (Huntleigh Health Care Ltd) in the supine position after a 10-min rest. ABI was calculated as the ratio of ankle systolic pressure divided by arm systolic pressure. Atherosclerosis in peripheral arteries was defined as ABI values of 0.9 or less [29].

### Systemic atherosclerosis

Systemic atherosclerosis was reflected by three indices, including the extent of atherosclerotic plaques, extent of atherosclerotic stenosis, and presence of PolyVD.

The extent of atherosclerotic plaques was defined according to the number of 8 vascular sites affected with plaques (intracranial, extracranial, coronary, subclavian, aorta, renal, iliofemoral or peripheral arteries), and further divided into four groups, including 0, 1, 2–3, or 4–8 vascular sites [30]. Similarly, the extent of atherosclerotic stenosis was defined according to the number of 8 vascular sites affected with stenosis, and divided into four groups (0, 1, 2–3, or 4–8 vascular sites) [30]. PolyVD was defined as the presence of atherosclerotic stenosis in at least two vascular sites of above 8 arteries [31].

### Statistical analysis

The baseline characteristic data of continuous variables were presented as mean (standard deviations) or median (interquartile ranges) and were compared by the analysis of variance or Wilcoxon rank sum test. The data of categorical variables were presented as frequencies (percentages) and were compared by Chi-square test.

Ordinary logistic regression analyses were performed to assess the associations of MAFLD and MAFLD subtypes with the extent of atherosclerotic plaques and stenosis, and to calculate common odds ratio (cOR) and 95% confidence intervals (95% CI). Binary logistic regression analyses were performed to assess the associations of MAFLD and MAFLD subtypes with the presence of PolyVD as well as the presence of atherosclerotic plaques and stenosis in a single vascular bed. Corresponding odds ratio (OR) and 95% CI were calculated. Demographics, lifestyle, family history of atherosclerotic cardiovascular disease (ASCVD), eGFR, and anti-atherosclerosis treatments were adjusted in models. To assess the robustness of the findings, sensitivity analyses were performed in following populations: participants without family history of ASCVD, without anti-atherosclerosis treatment, without smoking, and with eGFR ≥ 60 mL/min/1.73 m^2^, respectively. In addition, subgroup analyses were performed to determine whether the associations were consistent even in participants with different characteristics, including age, sex, and the status of alcohol consumption.

In addition, to determine the relationship between FLD and systemic atherosclerosis without the influence of MD in participants with different subtypes of MAFLD, we performed separate analyses in MAFLD participants with different types of MD using those having MD but without FLD as the reference. We further assessed the interaction of FLD and MD on systemic atherosclerosis using binary and ordinary logistic regression analyses. All statistical analyses were conducted using SAS version 9.4 (SAS Institute Inc, Cary, NC). A two-tailed *P* value < 0.05 was considered statistically significant.

## Results

### Baseline characteristics

The PRECISE study enrolled 3067 participants at baseline. We excluded 18 participants with malignancy and 2 participants without the data of height, weight, waist circumference, TG, and gamma-glutamyltransferase. Ultimately, 3047 participants were included in the final analyses (Additional file 1: Fig. S1). The mean age was 61.2 ± 6.7 years, and 46.6% (n = 1420) were male. The prevalence of MAFLD was 48.2% (n = 1469). The proportions for DM-MAFLD, OW-MAFLD, and lean-MAFLD were 29.0% (n = 426), 62.1% (n = 912), and 8.9% (n = 131), respectively. Participants with MAFLD were more likely to be male, had a higher prevalence of diabetes, hypertension, and dyslipidemia, and had a higher level of BMI than those with non-MAFLD (Table 1).

**Table 1:** Demographic and clinical characteristics of participants

Characteristic	Total (n = 3047)	Non-MAFLD (n = 1578)	MAFLD (n = 1469)	*P* value
Age, mean ± SD, year	61.2 ± 6.7	61.3 ± 6.7	61.1 ± 6.6	0.37
Male sex, n (%)	1420 (46.6)	665 (42.1)	755 (51.4)	< 0.001
Current smoking, n (%)	627 (20.6)	310 (19.7)	317 (21.6)	0.19
Current drinking, n (%)	574 (18.8)	254 (16.1)	320 (21.8)	< 0.001
Body mass index, mean ± SD, kg/m^2^	23.8 ± 3.0	21.9 ± 2.1	25.8 ± 2.6	< 0.001
Waist circumference, mean ± SD, cm	86.7 ± 8.9	81.1 ± 6.5	92.8 ± 6.9	< 0.001
Systolic blood pressure, mean ± SD, mmHg	129.3 ± 16.3	126.3 ± 16.4	132.5 ± 15.6	< 0.001
Diastolic blood pressure, mean ± SD, mmHg	75.2 ± 9.0	73.3 ± 8.7	77.3 ± 9.0	< 0.001
Platelet count, mean ± SD, × 10^9^/L	211.4 ± 56.9	209.1 ± 57.6	213.8 ± 56.1	0.02
AST, median (25th–75th), U/L	22.0 (19.0–26.0)	21.0 (18.0–25.0)	23.0 (19.0–28.0)	< 0.001
ALT, median (25th–75th), U/L	18.0 (14.0–26.0)	16.0 (13.0–21.0)	23.0 (17.0–31.0)	< 0.001
GGT, median (25th–75th), U/L	23.0 (16.0–35.0)	17.0 (14.0–23.0)	31.0 (23.0–51.0)	< 0.001
Total cholesterol, mean ± SD, mg/dL	203.9 ± 38.4	199.0 ± 37.0	209.1 ± 39.2	< 0.001
HDL-C, mean ± SD, mg/dL	52.7 ± 13.0	57.2 ± 13.1	47.9 ± 11.2	< 0.001
LDL-C, mean ± SD, mg/dL	107.2 ± 30.6	105.8 ± 29.3	108.6 ± 31.9	0.01
Triglycerides, median (25th–75th), mg/dL	129.2 (92.0–189.4)	100.0 (77.0–130.1)	176.1 (131.9–244.3)	< 0.001
Fasting blood glucose, mean ± SD, mmol/L	6.0 ± 1.6	5.7 ± 1.3	6.3 ± 1.8	< 0.001
HbA1c, mean ± SD, %	5.9 ± 0.9	5.8 ± 0.8	6.1 ± 1.1	< 0.001
eGFR, mean ± SD, mL/min/1.73 m^2^	101.9 ± 12.7	102.6 ± 11.8	101.2 ± 13.7	0.003
Uric acid, mean ± SD, μmol/L	340.6 ± 86.2	316.2 ± 75.7	366.7 ± 89.2	< 0.001
HOMA-IR, median (25th–75th)	1.6 (1.1–2.4)	1.3 (0.9–1.8)	2.1 (1.6–3.1)	< 0.001
Family history of ASCVD, n (%)	612 (20.1)	298 (18.9)	314 (21.4)	0.09
Diabetes mellitus, n (%)	658 (21.6)	232 (14.7)	426 (29.0)	< 0.001
Hypertension, n (%)	1311 (43.0)	514 (32.6)	797 (54.3)	< 0.001
Dyslipidemia, n (%)	1271 (41.7)	452 (28.6)	819 (55.8)	< 0.001
Concomitant medication, n (%)				
Antihypertensive	817 (26.8)	300 (19.0)	517 (35.2)	< 0.001
Lipid lowering	118 (3.9)	50 (3.2)	68 (4.6)	0.04
Antidiabetic	271 (8.9)	100 (6.3)	171 (11.6)	< 0.001
Antiplatelet	79 (2.6)	29 (1.8)	50 (3.4)	0.007
Anticoagulants	4 (0.1)	0 (0.0)	4 (0.3)	0.04

MAFLD, metabolic dysfunction-associated fatty liver disease; AST, aspartate aminotransferase; ALT, alanine transaminase; GGT, gamma-glutamyltransferase; HDL-C, high-density lipoprotein cholesterol; LDL-C, low-density lipoprotein cholesterol; HbA1c, glycosylated hemoglobin A1c; eGFR, estimated glomerular filtration rate; HOMA-IR, homeostasis model assessment-insulin resistance; ASCVD, atherosclerotic cardiovascular disease; SD, standard deviations

### Association of MAFLD with systemic atherosclerosis

**Fig. 1:**
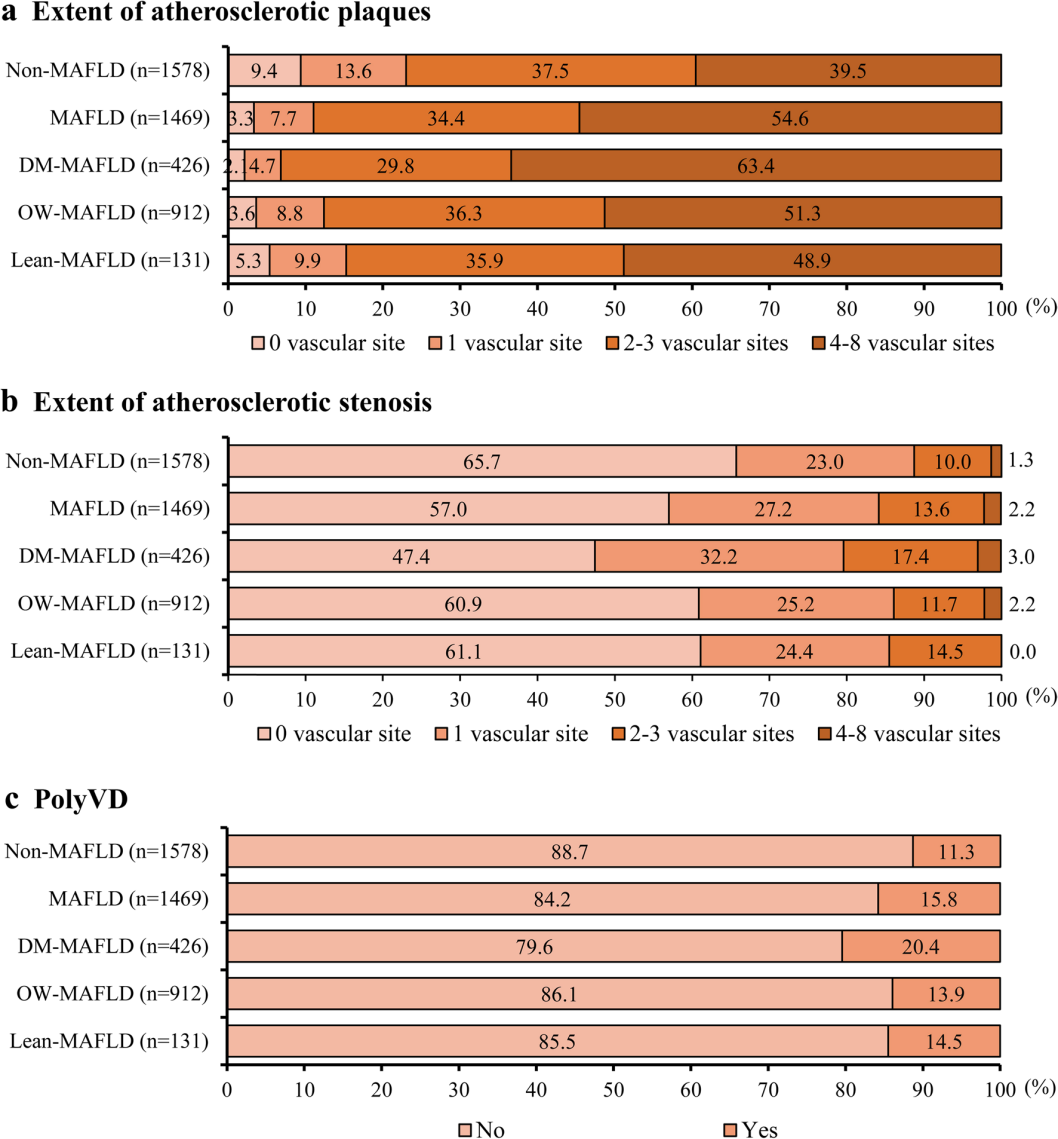
Distributions of atherosclerotic plaques and stenosis and presence of PolyVD in participants. MAFLD, metabolic dysfunction-associated fatty liver disease; PolyVD, polyvascular disease; DM, diabetes mellitus; OW, overweight or obesity. **a** Extent of atherosclerotic plaques. **b** Extent of atherosclerotic stenosis. **c** PolyVD

The atherosclerotic plaques, stenosis, and PolyVD were observed in 96.7%, 43.0%, and 15.8% of participants with MAFLD. In participants with DM-MAFLD, 97.9% had at least one-site atherosclerotic plaque, and 93.2% had multi-site atherosclerotic plaques, which were higher than the corresponding prevalence in OW-MAFLD and lean-MAFLD. Similar results were observed in individuals with atherosclerotic stenosis and PolyVD (Fig. 1).

After adjustment for demographics, lifestyle, family history of ASCVD, eGFR, and anti-atherosclerosis treatment, we found that MAFLD was associated with higher extent of atherosclerotic plaques (cOR, 2.14, 95% CI 1.85–2.48) and stenosis (cOR, 1.47, 95% CI 1.26–1.71), and higher odds of presence of PolyVD (OR, 1.55, 95% CI 1.24–1.94). In addition, DM-MAFLD and OW-MAFLD were associated with the extent of atherosclerotic plaques and stenosis, and presence of PolyVD (All *P* < 0.05). However, lean-MAFLD was only associated with the extent of atherosclerotic plaques (cOR, 1.63, 95% CI 1.14–2.34) (Table 2). After further analysis in individual vascular bed, we found that MAFLD was more closely associated with the atherosclerotic plaques in aorta arteries (OR, 2.17, 95% CI 1.78–2.65), iliofemoral arteries (OR, 2.09, 95% CI 1.73–2.53) and the atherosclerotic stenosis in subclavian arteries (OR, 1.53, 95% CI 1.20–1.96), iliofemoral arteries (OR, 1.72, 95% CI 1.40–2.11) (Additional file 1: Table S2).

**Table 2:** Association of MAFLD and MAFLD subtypes with systemic atherosclerosis

Factor	N	Extent of atherosclerotic plaques^a^		Extent of atherosclerotic stenosis^a^		PolyVD^b^	
		Adj. cOR (95% CI)	*P* value	Adj. cOR (95% CI)	*P* value	Adj. OR (95% CI)	*P* value
Non-MAFLD	1578	Ref.		Ref.		Ref.	
MAFLD	1469	2.14 (1.85–2.48)	< 0.001	1.47 (1.26–1.71)	< 0.001	1.55 (1.24–1.94)	< 0.001
DM-MAFLD	426	2.84 (2.25–3.57)	< 0.001	2.00 (1.61–2.47)	< 0.001	1.92 (1.42–2.60)	< 0.001
OW-MAFLD	912	1.97 (1.67–2.33)	< 0.001	1.29 (1.08–1.53)	0.004	1.39 (1.07–1.80)	0.01
Lean-MAFLD	131	1.63 (1.14–2.34)	0.007	1.18 (0.81–1.72)	0.38	1.36 (0.79–2.36)	0.27

MAFLD, metabolic dysfunction-associated fatty liver disease; PolyVD, polyvascular disease; DM, diabetes mellitus; OW, overweight or obesity; Adj., adjusted; cOR, common odds ratio; OR, odds ratio; CI, confidence intervals; Ref., reference

All models were adjusted for sex, age, marital status, education, income, current smoking, current drinking, sleep time, sedentary time, family history of ASCVD, eGFR, lipid-lowering medication, antiplatelet medication, and anticoagulants medication

^a^The extent of atherosclerotic plaques and stenosis was defined according to the number of 8 vascular sites affected and was divided into four groups, including 0, 1, 2–3, and 4–8 vascular sites. Adjusted cOR and 95% CI were calculated by the ordinary logistic regression model

^b^PolyVD was defined as the presence of atherosclerotic stenosis in at least two vascular sites. Adjusted OR and 95% CI were calculated by the binary logistic regression model

In sensitivity analyses, similar trends were observed in the participants without family history of ASCVD, without anti-atherosclerosis treatment, without smoking, and with eGFR ≥ 60 mL/min/1.73 m^2^ (Additional file 1: Table S3). Furthermore, we observed similar results across different characteristics of age, sex, and the status of alcohol consumption. All *P* values for interaction > 0.05 (Additional file 1: Fig. S2).

### Association of FLD with systemic atherosclerosis in participants with MAFLD

**Fig. 2:**
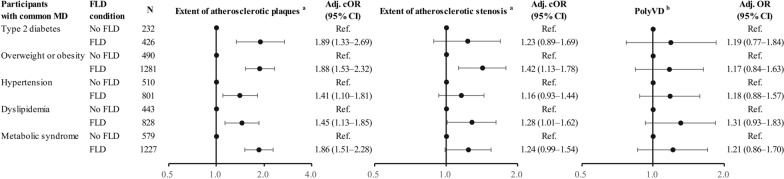
Association of FLD with systemic atherosclerosis in participants with common metabolic dysfunction. MD, metabolic dysfunction; FLD, fatty liver disease; PolyVD, polyvascular disease; cOR, common odds ratio; OR, odds ratio; CI, confidence intervals; Adj., adjusted; Ref., reference. All models were adjusted for sex, age, marital status, education, income, current smoking, current drinking, sleep time, sedentary time, family history of ASCVD, eGFR, lipid-lowering medication, antiplatelet medication, and anticoagulants medication. ^a^The extent of atherosclerotic plaques and stenosis was defined according to the number of 8 vascular sites affected and was divided into four groups, including 0, 1, 2–3, and 4–8 vascular sites. Adjusted cOR and 95% CI were calculated by the ordinary logistic regression model. ^b^PolyVD was defined as the presence of atherosclerotic stenosis in at least two vascular sites. Adjusted OR and 95% CI were calculated by the binary logistic regression model

Compared to those with MD but without FLD, we found that FLD was associated with the extent of atherosclerotic plaques in participants with T2DM (cOR, 1.89, 95% CI 1.33–2.69), overweight or obesity (cOR, 1.88, 95% CI 1.53–2.32), hypertension (cOR, 1.41, 95% CI 1.10–1.81), dyslipidemia (cOR, 1.45, 95% CI 1.13–1.85), and metabolic syndrome (cOR, 1.86, 95% CI 1.51–2.28). Additionally, FLD was associated with the extent of atherosclerotic stenosis in participants with overweight or obesity (cOR, 1.42, 95% CI 1.13–1.78), and dyslipidemia (cOR, 1.28, 95% CI 1.01–1.62). However, FLD was not associated with presence of PolyVD in participants with common MD (Fig. 2).

### Interaction of FLD and MD on systemic atherosclerosis

After adjusting for demographics, lifestyle, family history of ASCVD, eGFR, and anti-atherosclerosis treatment, we found that FLD and MD were associated with the extent of atherosclerotic plaques and stenosis, as well as presence of PolyVD compared with the absence of FLD and MD (All *P* < 0.05). Additionally, FLD combined with MD was associated with higher extent of atherosclerotic plaques (cOR, 3.28, 95% CI 2.67–4.02) and stenosis (cOR, 2.34, 95% CI 1.84–2.98), and higher odds of presence of PolyVD (OR, 2.87, 95% CI 1.91–4.33). The *P* values for interaction of the extent of plaques and stenosis, and presence of PolyVD were 0.055, 0.006 and 0.02, respectively (Table 3).

**Table 3:** Association of fatty liver disease, metabolic dysfunction and their interaction with systemic atherosclerosis

Factor	N	Extent of atherosclerotic plaques^a^		Extent of atherosclerotic stenosis^a^		PolyVD^b^	
		Adj. cOR (95% CI)	*P* value	Adj. cOR (95% CI)	*P* value	Adj. OR (95% CI)	*P* value
No FLD/No MD	474	Ref.		Ref.		Ref.	
No FLD/MD	1089	1.82 (1.48–2.24)	< 0.001	1.88 (1.46–2.42)	< 0.001	2.24 (1.46–3.43)	< 0.001
FLD/No MD	20	4.47 (1.78–11.21)	0.001	4.30 (1.80–10.24)	0.001	5.57 (1.65–18.76)	0.006
FLD/MD	1464	3.28 (2.67–4.02)	< 0.001	2.34 (1.84–2.98)	< 0.001	2.87 (1.91–4.33)	< 0.001
*P* for interaction			0.055		0.006		0.02

MD, metabolic dysfunction; FLD, fatty liver disease; PolyVD, polyvascular disease; cOR, common odds ratio; OR, odds ratio; CI, confidence intervals; Adj., adjusted; Ref., reference

All models were adjusted for sex, age, marital status, education, income, current smoking, current drinking, sleep time, sedentary time, family history of ASCVD, eGFR, lipid-lowering medication, antiplatelet medication, and anticoagulants medication

^a^The extent of atherosclerotic plaques and stenosis was defined according to the number of 8 vascular sites affected and was divided into four groups, including 0, 1, 2–3, and 4–8 vascular sites. Adjusted cOR and 95% CI were calculated by the ordinary logistic regression model

^b^PolyVD was defined as the presence of atherosclerotic stenosis in at least two vascular sites. Adjusted OR and 95% CI were calculated by the binary logistic regression model

## Discussion

In this largescale community-dwelling study, we found that MAFLD was associated with the extent of atherosclerotic plaques and stenosis, and presence of PolyVD. We also observed the associations of its subtypes of DM-MAFLD and OW-MAFLD with systemic atherosclerosis. Furthermore, as one component of MAFLD, FLD per se was associated with the extent of atherosclerotic plaques and stenosis. Notably, FLD interacted with MD to increase the odds of presence of systemic atherosclerosis in participants with MAFLD.

MAFLD is a novel terminology redefined from non-alcoholic fatty liver disease and highlights the coexistence of MD and FLD [6]. It is well established that MD, such as dyslipidemia, diabetes mellitus, and hypertension, is a prominent contributor to the development of atherosclerosis [1]. In addition, FLD has also been reported to damage the structures and functions of vascular and promote the development of atherosclerosis by damaging vascular endothelial cell, activating the coagulation system, and increasing atherogenic lipid levels [32, 33]. This may be a potential mechanism of the association between MAFLD and systemic atherosclerosis. A cross-sectional study with 890 patients reported the association between MAFLD and subclinical atherosclerosis in coronary and carotid arteries [12]. Most importantly, recent cohort studies conducted in community-dwelling residents showed that patients with long-term MAFLD had increased odds and risk for atherosclerosis development in coronary, carotid and peripheral arteries [11, 13, 34]. However, previous studies focused on elevated carotid intima-media thickness and brachial-ankle pulse wave velocity [11, 13], which merely reflect the alteration of vasculature and arterial elasticity [35, 36]. In our study, given the atherosclerotic systematicity, we assessed the association between MAFLD and systemic atherosclerosis, which further included intracranial, extracranial, renal, and iliofemoral arterial territories. Consequently, our study may provide more robust and elaborate evidence on the association between MAFLD and systemic atherosclerosis. Additionally, our findings highlighted the importance of the identification and management of MAFLD at an early stage for the prevention of systemic atherosclerosis.

MD is the main distinction among MAFLD subtypes. Although multiple MD factors involved in the development of atherosclerosis, insulin resistance may play a crucial role compared with other risk factors. Insulin resistance in the liver and muscle causes the onset of T2DM and induces other atherosclerotic conditions such as obesity, hypertension, dyslipidemia, and hyperinsulinemia [37, 38]. The pathogenic mechanisms linking insulin resistance to atherosclerosis include proinflammatory state, perturbation of insulin signaling at the level of the intimal cells, and induction of other metabolic conditions [39]. As T2DM and obesity are common diseases associated with high insulin resistance [40], this might explain why DM-MAFLD and OW-MAFLD were associated with atherosclerotic plaques and stenosis in our study. Meanwhile, those findings indicated that MAFLD patients with T2DM or obesity may desire more attention in clinical practice to prevent the poor clinical outcomes of systemic atherosclerosis.

Although FLD has been reported to promote the development of atherosclerosis [32, 33], it remains in debate whether FLD independently contributes to atherosclerosis or the association between FLD and atherosclerosis is just a reflection of shared comorbidities [3]. In this study, by using the participant with MD but without FLD as reference, we demonstrated that FLD was associated with higher extent of atherosclerotic plaques and stenosis. Notably, we additionally demonstrated that FLD interacted with MD to promote the prevalence of systemic atherosclerosis. These results indicated that FLD per se may be an independent risk for atherosclerosis. These findings also implicated that FLD might be a potential target of intervention for prevention of atherosclerosis in participants with MAFLD.

The major strength of this study is that we comprehensively assessed multi-site atherosclerosis using advanced vascular imaging techniques in a largescale community-based population. Nevertheless, there are still several limitations. First, it is not warranted to infer causal relationships due to the cross-sectional study design of the study. More high-quality longitudinal studies are needed to verify the relationship between MAFLD and systemic atherosclerosis. Second, despite representative as a whole, potential selection bias existed in this study because the participants were enrolled from a single community region. Third, participants in our study were restricted to Chinese elderly adults, thus it should be cautious to extrapolate the findings to other populations. The findings of current study need to be further validated in a larger and more ethnically diverse cohort.

In conclusion, MAFLD and its subtypes of DM-MAFLD and OW-MAFLD were associated with higher extent of atherosclerotic plaques and stenosis, and presence of PolyVD in a community-based population. FLD interacted with MD to increase the odds of presence of systemic atherosclerosis in participants with MAFLD. This study indicated the potentially deleterious effects of MAFLD on systemic atherosclerosis and implicated that FLD might be an intervention target for the prevention of atherosclerosis in participants with MAFLD.

### Supplementary Information

**Additional file 1:** **Table S1.** Definitions of fatty liver disease, MAFLD, MAFLD subtypes, no metabolic dysfunction, and metabolic syndrome. **Table S2.** Association of MAFLD and MAFLD subtypes with atherosclerosis in a single vascular bed. **Table S3.** Sensitivity analysis on the association of MAFLD and MAFLD subtypes with systemic atherosclerosis. **Figure S1.** Flowchart of the study. **Figure S2.** Association of MAFLD, MAFLD subtypes with systemic atherosclerosis in participants with different characteristics.

## Data Availability

The data underlying this article will be shared on reasonable request to the corresponding author.

## Abbreviations

ABI: Ankle-brachial index
ASCVD: Atherosclerotic cardiovascular disease
BMI: Body mass index
cOR: Common odds ratio
CTA: Computed tomography angiography
eGFR: Estimated glomerular filtration rate
FBG: Fasting blood glucose
FLD: Fatty liver disease
HbA1c: Glycosylated hemoglobin A1c
HDL-C: High-density lipoprotein cholesterol
HOMA-IR: Homeostatic model assessment-insulin resistance
MAFLD: Metabolic dysfunction-associated fatty liver disease
MD: Metabolic dysfunction
MRI: Magnetic resonance imaging
LDL-C: Low-density lipoprotein cholesterol
OR: Odds ratio
PolyVD: Polyvascular disease
PRECISE: Polyvascular Evaluation for Cognitive Impairment and Vascular Events
TC: Total cholesterol
TG: Triglycerides
T2DM: Type 2 diabetes mellitus
