# Supplementary material for: Association of metabolic dysfunction-associated fatty liver disease with systemic atherosclerosis: a community-based cross-sectional study
Source: Cardiovasc Diabetol. 2023 Dec 13;22:342. doi: 10.1186/s12933-023-02083-0 (PMC10720122; doi:10.1186/s12933-023-02083-0)
Supplement: Supplementary file 1 — Additional file 1: Table S1. Definitions of fatty liver disease, MAFLD, MAFLD subtypes, no metabolic dysfunction, and metabolic syndrome. Table S2. Association of MAFLD and MAFLD subtypes with atherosclerosis in a single vascular bed. Table S3. Sensitivity analysis on the association of MAFLD and MAFLD subtypes with systemic atherosclerosis. Figure S1. Flowchart of the study. Figure S2. Association of MAFLD, MAFLD subtypes with systemic atherosclerosis in participants with different characteristics. [file 12933_2023_2083_MOESM1_ESM.docx]

TABLES

Table S1 Definitions of fatty liver disease, MAFLD, MAFLD subtypes, no metabolic dysfunction, and metabolic syndrome

| Definition | Criteria |
| --- | --- |
| Fatty liver disease [1] | FLI ≥30; FLI = e^x^ / (1 + e^x^) × 100, where x = 0.953 × log_e_ triglyceride + 0.139 × body mass index + 0.718 × log_e_ gamma-glutamyltransferase + 0.053 × waist circumference − 15.745 |
|  |  |
| MAFLD [2] | The presence of fatty liver disease plus one or more of the following conditions: 1. overweight or obesity (body mass index ≥23 kg/m^2^ in Asians); 2. type 2 diabetes mellitus; 3. lean or normal weight (body mass index <23 kg/m^2^ in Asians) but the presence of at least two metabolic abnormalities, including  a. waist circumference ≥90 cm in Asian men and ≥80 cm in Asian women; b. blood pressure ≥130/85 mmHg or specific drug treatment; c. triglycerides ≥150 mg/dL or specific drug treatment; d. high-density lipoprotein cholesterol <40 mg/dL in men and <50 mg/dL in women or specific drug treatment; e. prediabetes;  f. homeostasis model assessment-insulin resistance score ≥2.5; g. plasma high-sensitivity C-reactive protein >2 mg/L. |
|  |  |
| DM-MAFLD | The presence of fatty liver disease plus type 2 diabetes mellitus. |
|  |  |
| OW-MAFLD | Without type 2 diabetes mellitus, but the presence of fatty liver disease plus overweight or obesity. |
|  |  |
| Lean-MAFLD | Without type 2 diabetes mellitus, overweight or obesity, but the presence of fatty liver disease plus at least two metabolic abnormalities listed below: a. waist circumference ≥90 cm in Asian men and ≥80 cm in Asian women; b. blood pressure ≥130/85 mmHg or specific drug treatment; c. triglycerides ≥150 mg/dL or specific drug treatment; d. high-density lipoprotein cholesterol <40 mg/dL in men and <50 mg/dL in women or specific drug treatment; e. prediabetes;  f. homeostasis model assessment-insulin resistance score ≥2.5; g. plasma high-sensitivity C-reactive protein >2 mg/L. |
|  |  |
| No metabolic dysfunction | Without type 2 diabetes mellitus, overweight or obesity, and less than two metabolic abnormalities listed below: a. waist circumference ≥90 cm in Asian men and ≥80 cm in Asian women; b. blood pressure ≥130/85 mmHg or specific drug treatment; c. triglycerides ≥150 mg/dL or specific drug treatment; d. high-density lipoprotein cholesterol <40 mg/dL in men and <50 mg/dL in women or specific drug treatment; e. prediabetes;  f. homeostasis model assessment-insulin resistance score ≥2.5; g. plasma high-sensitivity C-reactive protein >2 mg/L. |
|  |  |
| Metabolic syndrome [3] | The presence of central obesity (waist circumference ≥ 90 cm for South and East Asian men and ≥ 80 cm for South and East Asian women) plus any two of the following conditions: 1. raised triglycerides: ≥150 mg/dL or specific treatment for this lipid abnormality; 2. reduced high-density lipoprotein cholesterol: <40 mg/dL in males or <50 mg/dL in females or specific treatment for this lipid abnormality; 3. raised blood pressure: ≥130/85 mmHg or treatment of previously identified hypertension; 4. raised fasting glucose: ≥100 mg/dL or previously diagnosed type 2 diabetes mellitus. |

Abbreviations: MAFLD, metabolic dysfunction-associated fatty liver disease; FLI, fatty liver index; DM, diabetes mellitus; OW, overweight or obesity.

References

1. Han AL. Validation of fatty liver index as a marker for metabolic dysfunction-associated fatty liver disease. Diabetol Metab Syndr. 2022;14(1):44. doi:10.1186/s13098-022-00811-2

2. Eslam M, Sarin SK, Wong VW, et al. The Asian Pacific Association for the study of the liver clinical practice guidelines for the diagnosis and management of metabolic associated fatty liver disease. Hepatol Int. 2020;14(6):889-919. doi:10.1007/s12072-020-10094-2

3. Zhu L, Spence C, Yang JW, Ma GX. The IDF definition is better suited for screening metabolic syndrome and estimating risks of diabetes in Asian American adults: Evidence from NHANES 2011-2016. J Clin Med. 2020;9(12):3871. doi:10.3390/jcm9123871

## Table S2 Association of MAFLD and MAFLD subtypes with atherosclerosis in a single vascular bed

| Vascular bed | Factor | Presence of atherosclerotic plaques ^a^ | | |  | Presence of atherosclerotic stenosis ^b^ | | |
| --- | --- | --- | --- | --- | --- | --- | --- | --- |
|  |  | Case/N | Adj. OR (95%CI) | *P* value |  | Case/N | Adj. OR (95%CI) | *P* value |
| Intracranial arteries | Non-MAFLD | 234/1577 | Ref. |  |  | 67/1577 | Ref. |  |
|  | MAFLD | 303/1468 | 1.53 (1.26–1.85) | <0.001 |  | 75/1468 | 1.23 (0.87–1.73) | 0.25 |
|  | DM-MAFLD | 119/425 | 2.14 (1.65–2.77) | <0.001 |  | 30/425 | 1.56 (0.99–2.46) | 0.06 |
|  | OW-MAFLD | 169/912 | 1.36 (1.09–1.70) | 0.007 |  | 42/912 | 1.14 (0.77–1.71) | 0.51 |
|  | Lean-MAFLD | 15/131 | 0.81 (0.46–1.42) | 0.46 |  | 3/131 | 0.58 (0.18–1.88) | 0.36 |
| Extracranial arteries | Non-MAFLD | 540/1573 | Ref. |  |  | 17/1573 | Ref. |  |
|  | MAFLD | 562/1456 | 1.11 (0.96–1.30) | 0.17 |  | 16/1456 | 0.93 (0.46–1.89) | 0.85 |
|  | DM-MAFLD | 157/423 | 1.03 (0.82–1.29) | 0.81 |  | 11/423 | 2.20 (0.99–4.84) | 0.051 |
|  | OW-MAFLD | 347/902 | 1.12 (0.94–1.33) | 0.22 |  | 5/902 | 0.47 (0.17–1.31) | 0.15 |
|  | Lean-MAFLD | 58/131 | 1.43 (0.99–2.06) | 0.06 |  | 0/131 | – | – |
| Subclavian arteries | Non-MAFLD | 692/1553 | Ref. |  |  | 129/1553 | Ref. |  |
|  | MAFLD | 796/1438 | 1.62 (1.38–1.89) | <0.001 |  | 175/1438 | 1.53 (1.20–1.96) | <0.001 |
|  | DM-MAFLD | 259/418 | 1.93 (1.53–2.45) | <0.001 |  | 45/418 | 1.17 (0.81–1.69) | 0.40 |
|  | OW-MAFLD | 463/893 | 1.46 (1.22–1.75) | <0.001 |  | 120/893 | 1.82 (1.39–2.39) | <0.001 |
|  | Lean-MAFLD | 74/127 | 1.87 (1.26–2.76) | 0.002 |  | 10/127 | 1.00 (0.50–1.98) | 0.99 |
| Coronary arteries | Non-MAFLD | 611/1572 | Ref. |  |  | 253/1572 | Ref. |  |
|  | MAFLD | 742/1453 | 1.64 (1.40–1.91) | <0.001 |  | 286/1453 | 1.26 (1.03–1.53) | 0.02 |
|  | DM-MAFLD | 247/422 | 2.03 (1.61–2.55) | <0.001 |  | 111/422 | 1.70 (1.30–2.22) | <0.001 |
|  | OW-MAFLD | 436/901 | 1.52 (1.27–1.81) | <0.001 |  | 154/901 | 1.08 (0.86–1.36) | 0.49 |
|  | Lean-MAFLD | 59/130 | 1.36 (0.93–1.98) | 0.12 |  | 21/130 | 1.04 (0.63–1.71) | 0.89 |
| Renal arteries | Non-MAFLD | 400/1569 | Ref. |  |  | 66/1569 | Ref. |  |
|  | MAFLD | 466/1456 | 1.45 (1.22–1.73) | <0.001 |  | 78/1456 | 1.19 (0.84–1.68) | 0.34 |
|  | DM-MAFLD | 154/423 | 1.49 (1.16–1.92) | 0.002 |  | 28/423 | 1.31 (0.81–2.12) | 0.27 |
|  | OW-MAFLD | 278/903 | 1.48 (1.21–1.80) | <0.001 |  | 45/903 | 1.17 (0.78–1.75) | 0.45 |
|  | Lean-MAFLD | 34/130 | 1.17 (0.76–1.82) | 0.48 |  | 5/130 | 0.87 (0.34–2.27) | 0.78 |
| Aorta arteries | Non-MAFLD | 1155/1564 | Ref. |  |  | 27/1553 | Ref. |  |
|  | MAFLD | 1248/1454 | 2.17 (1.78–2.65) | <0.001 |  | 38/1438 | 1.55 (0.93–2.58) | 0.10 |
|  | DM-MAFLD | 387/422 | 3.72 (2.55–5.43) | <0.001 |  | 14/418 | 1.91 (0.97–3.75) | 0.06 |
|  | OW-MAFLD | 754/902 | 1.89 (1.51–2.37) | <0.001 |  | 21/893 | 1.41 (0.78–2.55) | 0.26 |
|  | Lean-MAFLD | 107/130 | 1.57 (0.96–2.59) | 0.07 |  | 3/127 | 1.29 (0.38–4.45) | 0.69 |
| Iliofemoral arteries | Non-MAFLD | 1089/1572 | Ref. |  |  | 218/1572 | Ref. |  |
|  | MAFLD | 1206/1458 | 2.09 (1.73–2.53) | <0.001 |  | 308/1458 | 1.72 (1.40–2.11) | <0.001 |
|  | DM-MAFLD | 373/425 | 2.90 (2.08–4.03) | <0.001 |  | 126/425 | 2.69 (2.04–3.55) | <0.001 |
|  | OW-MAFLD | 733/903 | 1.96 (1.58–2.44) | <0.001 |  | 151/903 | 1.30 (1.02–1.66) | 0.04 |
|  | Lean-MAFLD | 100/130 | 1.39 (0.87–2.20) | 0.17 |  | 31/130 | 1.81 (1.13–2.91) | 0.01 |
| Peripheral arteries | Non-MAFLD | 29/1565 | Ref. |  |  | 29/1565 | Ref. |  |
|  | MAFLD | 20/1462 | 0.79 (0.44–1.42) | 0.42 |  | 20/1462 | 0.79 (0.44–1.42) | 0.42 |
|  | DM-MAFLD | 4/422 | 0.56 (0.19–1.63) | 0.29 |  | 4/422 | 0.56 (0.19–1.63) | 0.29 |
|  | OW-MAFLD | 16/909 | 1.01 (0.54–1.88) | 0.99 |  | 16/909 | 1.01 (0.54–1.88) | 0.99 |
|  | Lean-MAFLD | 0/131 | – | – |  | 0/131 | – | – |

Abbreviations: MAFLD, metabolic dysfunction-associated fatty liver disease; DM, diabetes mellitus; OW, overweight or obesity; OR, odds ratio; CI, confidence intervals; Adj., adjusted; Ref., reference.

All models were adjusted for sex, age, marital status, education, income, current smoking, current drinking, sleep time, sedentary time, family history of ASCVD, eGFR, lipid-lowering medication, antiplatelet medication, and anticoagulants medication. Adjusted OR and 95%CI were calculated by the binary logistic regression model.

^a^ Presence of atherosclerotic plaques in intracranial and extracranial arteries was defined as eccentric wall thickening with or without luminal stenosis, in coronary, subclavian, aorta, renal, and iliofemoral arteries was defined as tissue structures of at least one square millimeter area within or adjacent to the artery lumen and discernable from the vessel lumen, in peripheral arteries was defined as ABI values of 0.9 or less.

^b^ Presence of atherosclerotic stenosis in intracranial, extracranial, coronary, subclavian, aorta, renal, and iliofemoral arteries was defined as 50-99% of stenosis or occlusion, in peripheral arteries was defined as ABI values of 0.9 or less.

## Table S3 Sensitivity analysis on the association of MAFLD and MAFLD subtypes with systemic atherosclerosis

| Outcome | MAFLD | |  | MAFLD subtypes | | | |
| --- | --- | --- | --- | --- | --- | --- | --- |
|  | No | Yes |  | Non-MAFLD | DM-MAFLD | OW-MAFLD | Lean-MAFLD |
| Participants without family of ASCVD ^a^ | | |  |  |  |  |  |
| Extent of atherosclerotic plaques | Ref. | 2.03 (1.72–2.39) |  | Ref. | 2.59 (2.00–3.34) | 1.89 (1.57–2.28) | 1.54 (1.02–2.33) |
| Extent of atherosclerotic stenosis | Ref. | 1.48 (1.25–1.76) |  | Ref. | 1.95 (1.54–2.49) | 1.31 (1.08–1.60) | 1.24 (0.81–1.89) |
| PolyVD | Ref. | 1.58 (1.23–2.02) |  | Ref. | 2.01 (1.44–2.81) | 1.39 (1.04–1.86) | 1.40 (0.76–2.57) |
| Participants without anti-atherosclerosis treatment ^b^ | | | | |  |  |  |
| Extent of atherosclerotic plaques | Ref. | 2.15 (1.85–2.49) |  | Ref. | 2.71 (2.10–3.50) | 1.88 (1.56–2.27) | 1.50 (0.99–2.26) |
| Extent of atherosclerotic stenosis | Ref. | 1.44 (1.24–1.68) |  | Ref. | 1.99 (1.59–2.49) | 1.27 (1.06–1.52) | 1.16 (0.80–1.70) |
| PolyVD | Ref. | 1.53 (1.21–1.94) |  | Ref. | 1.90 (1.38–2.63) | 1.37 (1.05–1.80) | 1.44 (0.83–2.50) |
| Participants without smoking ^c^ | |  |  |  |  |  |  |
| Extent of atherosclerotic plaques | Ref. | 2.20 (1.84–2.64) |  | Ref. | 2.99 (2.25–3.97) | 2.03 (1.65–2.50) | 1.45 (0.90–2.33) |
| Extent of atherosclerotic stenosis | Ref. | 1.46 (1.20–1.77) |  | Ref. | 1.83 (1.39–2.41) | 1.30 (1.04–1.64) | 1.17 (0.67–2.06) |
| PolyVD | Ref. | 1.63 (1.19–2.25) |  | Ref. | 1.75 (1.15–2.67) | 1.54 (1.06–2.24) | 1.81 (0.76–4.33) |
| Participants with eGFR ≥60 mL/min/1.73 m^2 d^ | | |  |  |  |  |  |
| Extent of atherosclerotic plaques | Ref. | 2.16 (1.86–2.49) |  | Ref. | 2.84 (2.25–3.58) | 1.99 (1.68–2.35) | 1.67 (1.16–2.39) |
| Extent of atherosclerotic stenosis | Ref. | 1.47 (1.26–1.71) |  | Ref. | 1.98 (1.60–2.46) | 1.29 (1.09–1.54) | 1.19 (0.82–1.74) |
| PolyVD | Ref. | 1.55 (1.24–1.94) |  | Ref. | 1.93 (1.42–2.61) | 1.39 (1.07–1.81) | 1.36 (0.79–2.36) |

Abbreviations: MAFLD, metabolic dysfunction-associated fatty liver disease; PolyVD, polyvascular disease; ASCVD, atherosclerotic cardiovascular disease; eGFR estimated glomerular filtration rate; DM, diabetes mellitus; OW, overweight or obesity.

The extent of atherosclerotic plaques and stenosis was defined according to the number of 8 vascular sites affected and was divided into four groups, including 0, 1, 2-3, and 4-8 vascular sites. Adjusted common odds ratio and 95% confidence intervals were calculated by the ordinary logistic regression model.

PolyVD was defined as the presence of atherosclerotic stenosis in at least two vascular sites. Adjusted odds ratio and 95% confidence intervals were calculated by the binary logistic regression model.

Adjusted covariates included sex, age, marital status, education, income, current smoking, current drinking, sleep time, sedentary time, family history of ASCVD, eGFR, lipid-lowering medication, antiplatelet medication, and anticoagulants medication.

^a^ Adjusted for above covariates except family history of ASCVD.
^b^ Adjusted for above covariates except lipid-lowering medication, antiplatelet medication, and anticoagulants medication.
^c^ Adjusted for above covariates except current smoking.
^d^ Adjusted for above covariates except eGFR.

FIGURES


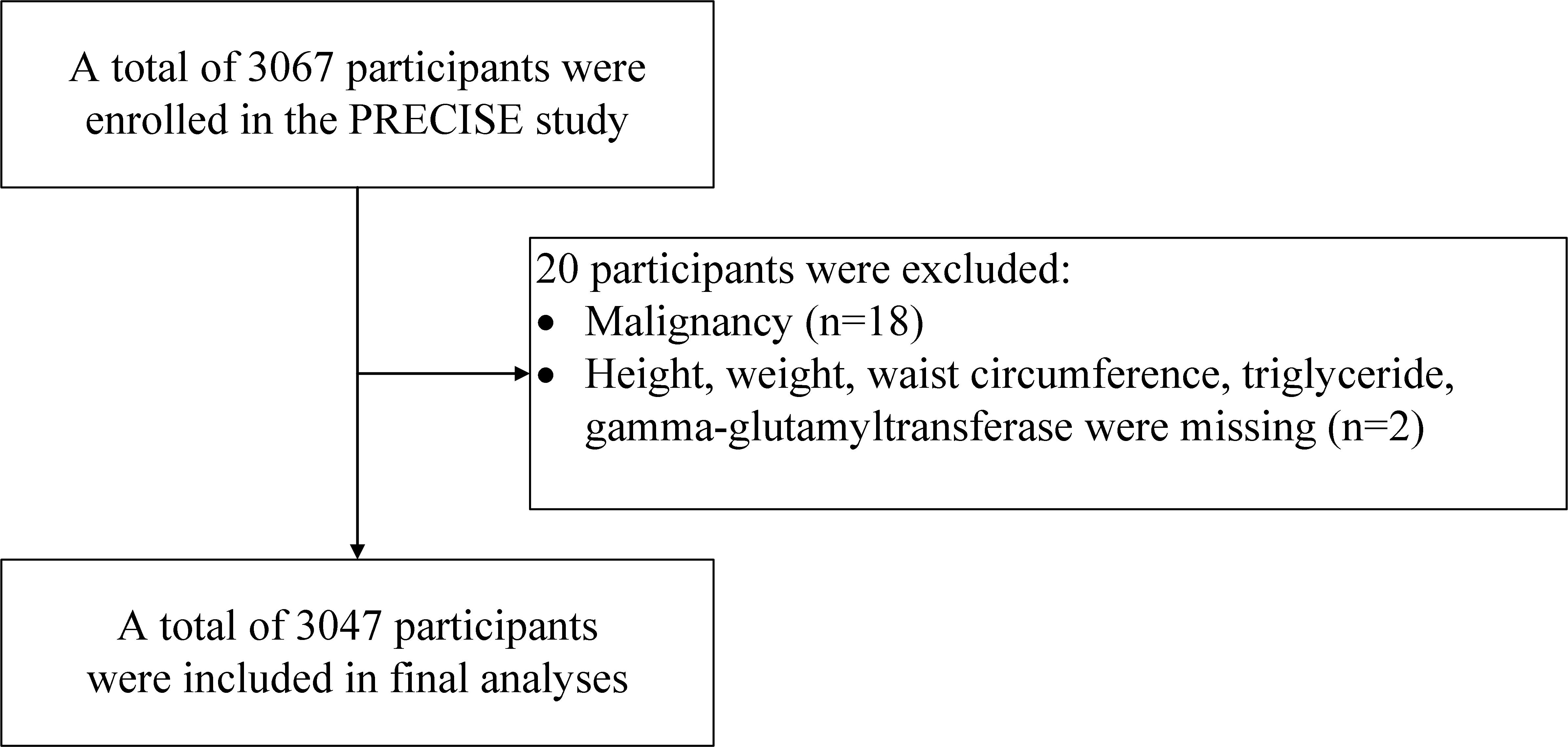


Fig. S1 Flowchart of the study

Abbreviations: PRECISE, Polyvascular Evaluation for Cognitive Impairment and Vascular Events


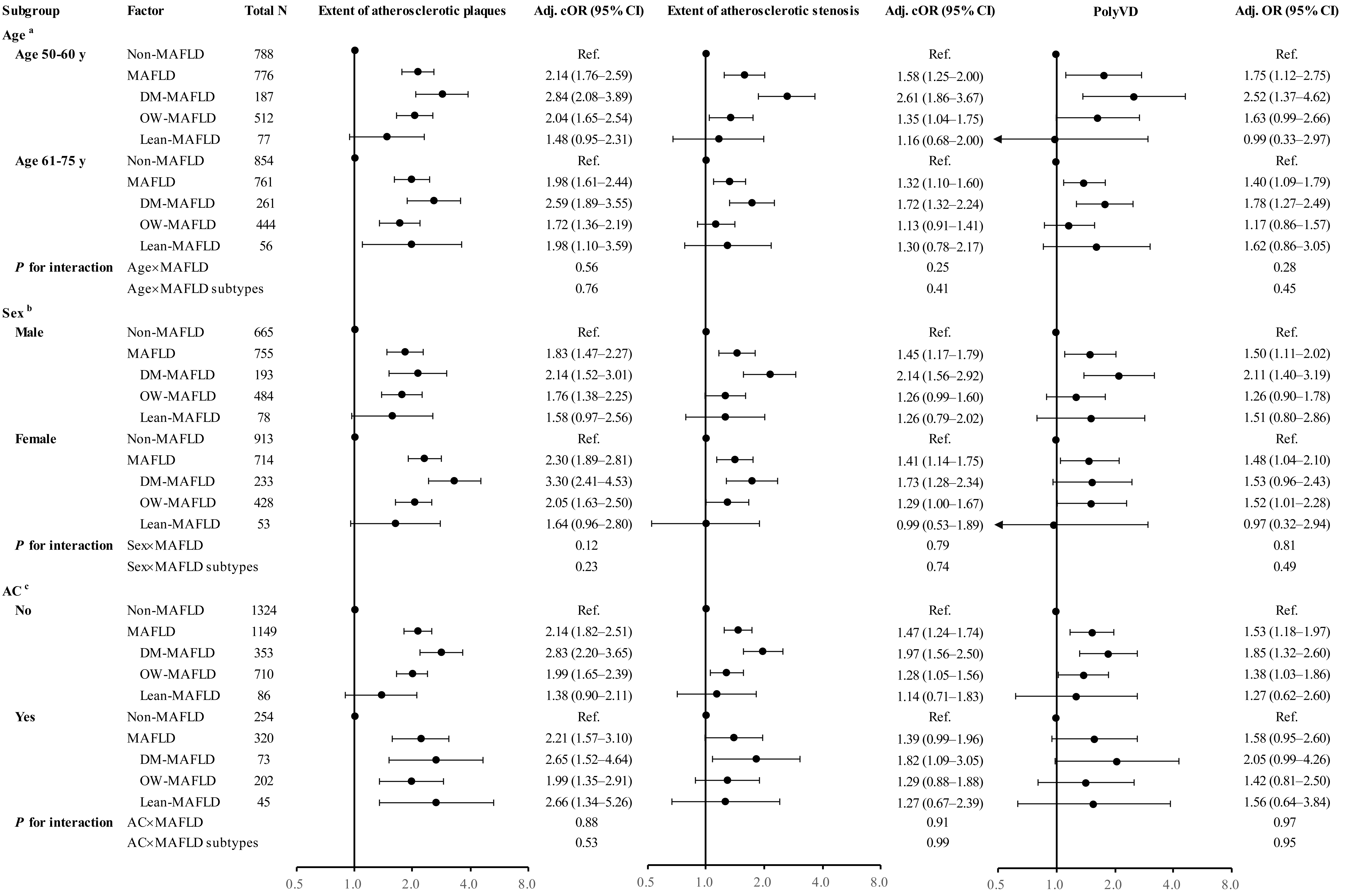


Fig. S2 Association of MAFLD, MAFLD subtypes with systemic atherosclerosis in participants with different characteristics

Abbreviations: MAFLD, metabolic dysfunction-associated fatty liver disease; PolyVD, polyvascular disease; cOR, common odds ratio; OR, odds ratio; CI, confidence intervals; DM, diabetes mellitus; OW, overweight or obesity; AC, alcohol consumption; Adj., adjusted; Ref., reference.

The extent of atherosclerotic plaques and stenosis was defined according to the number of 8 vascular sites affected and was divided into four groups, including 0, 1, 2-3, and 4-8 vascular sites. Adjusted cOR and 95%CI were calculated by the ordinary logistic regression model.

PolyVD was defined as the presence of atherosclerotic stenosis in at least two vascular sites. Adjusted OR and 95%CI were calculated by the binary logistic regression model.

Covariates included sex, age, marital status, education, income, current smoking, current drinking, sleep time, sedentary time, family history of atherosclerotic cardiovascular disease, eGFR, lipid-lowering medication, antiplatelet medication, and anticoagulants medication.

^a^ Adjusted for above covariates except age.

^b^ Adjusted for above covariates except sex.

^c^ Adjusted for above covariates except current drinking.
